# Ameliorative effects of quercetin against hepatic toxicity of oral sub-chronic co-exposure to aluminum oxide nanoparticles and lead-acetate in male rats

**DOI:** 10.1007/s00210-022-02351-y

**Published:** 2022-12-06

**Authors:** Khaled Abo-EL-Sooud, Yasmina M. Abd-Elhakim, Mohamed M. M. Hashem, Abeer E. El-Metwally, Bayan A. Hassan, Hayat H. M. El-Nour

**Affiliations:** 1 Department of Pharmacology, Faculty of Veterinary Medicine, Cairo University, Giza, Egypt; 2 Department of Forensic Medicine and Toxicology, Faculty of Veterinary Medicine, Zagazig University, Zagazig, Egypt; 3 Pathology Department, Animal Reproduction Research Institute, Giza, Egypt; 4 Pharmacology Department, Faculty of Pharmacy, Future University, Cairo, Egypt; 5 Biology of Reproduction Department, Animal Reproduction Research Institute, Giza, Egypt

**Keywords:** Aluminum oxide nanoparticles, Lead acetate, Quercetin, Liver, Oxidative stress

## Abstract

The present study was designed to evaluate the probable ameliorative role of quercetin (QCN) against oxidative hepatotoxicity induced by aluminum oxide nanoparticles (Al_2_O_3_NPs) with a diameter < 30 nm and lead acetate (Pb) co-exposure in adult male Sprague–Dawley rats. Rats were weighed and allocated to seven groups (*n* = 10 each) and were treated orally via orogastric gavage for 60 successive days: rats of the 1st group were kept as control given distilled water (1 ml/kg), rats of the 2nd group received 2 ml/kg BW/day corn oil; rats of the 3rd group were administered 20 mg/kg BW QCN/day; rats of the 4th group received 100 mg/kg BW Al_2_O_3_NPs; rats of the 5th group received 50 mg/kg BW Pb; rats of the 6th group co-received Al_2_O_3_NPs and Pb at the same previous doses; and rats of the 7th group were co-administered Al_2_O_3_NPs, Pb, and QCN at the same previous doses. At the end of the experiment, serum levels of alkaline phosphatase (ALP), alanine aminotransferase (ALT), aspartate aminotransferase (AST), total, direct, indirect bilirubin, triglycerides, total cholesterol, HDL, VLDL, and LDL were estimated. The hepatic oxidative stress biomarkers as superoxide dismutase (SOD), malondialdehyde (MDA), and glutathione peroxidase (GPx), were also evaluated. Finally, the histopathological and histomorphometric evaluations and the residues of Al and Pb in hepatic tissues were assessed. Al_2_O_3_NPs and/or Pb exposure significantly elevated lipid peroxidation levels and considerably altered the hepatic biochemical parameters; nevertheless, QCN significantly reduced hepatic enzymes compared to toxicant exposed groups. Additionally, QCN significantly improved Al_2_O_3_NPs-afforded liver tissue damage, as established in microscopic findings on the liver in the group treated with Al_2_O_3_NPs + Pb. Conclusively, QCN could be a candidate natural agent to safeguard the liver versus the co-harmful impacts of Al_2_O_3_NPs and Pb toxicity.

## Introduction

In this era, the widespread use of nanomaterial-containing products has increased the number of nanoparticles (NPs) released into the environment. Different sources of other pollutants frequently co-exist simultaneously with NPs as heavy metals (HMs) that are extensively distributed environmental pollutants (Abd-Elhakim et al. 2021). The interactions between NPs and HMs, as well as the organisms, could considerably impact complex systems. Aluminum oxide nanoparticles (Al_2_O_3_NPs) have been involved in many manufacturing purposes, including packaging materials, cutting tools, refractory products, cosmetic fillers, and semiconductor materials (Yousef et al. 2019). The Al_2_O_3_-NPs exposure considerably raised ROS release, while the hepatic-reduced glutathione (GSH) levels, catalase, and superoxide dismutase (SOD) activities were decreased (Shrivastava et al. 2014). Suppression of hepatic expression of peroxisome proliferator-activated receptor gamma-coactivator 1α (PGC-1α) and mitochondrial transcription factor A (mtTFA) gene was also confirmed with Al_2_O_3_-NPs exposure (Yousef et al. 2019). Moreover, significant alterations in lipid peroxidation (LPO) levels and biochemical parameters in the liver and testis were verified in male rats exposed to Al_2_O_3_-NPs (Hamza et al. 2018).

Lead (Pb) is concerned with hepatic disorders and significantly elevated hepatic transaminases, lipid profiles, oxidative stress biomarkers SOD, glutathione peroxidase (GPx), and total GSH with significant histopathological alterations (Offor et al. 2017; Albishtue et al. 2020). Al_2_O_3_NPs possibly exist as a pollutant in food and can enhance the bioavailability of Pb and some amendments to its toxic effect. The co-administration of Al_2_O_3_NPs and Pb increased the hepatic Pb-accumulation (Shumakova et al. 2015).

Quercetin (QCN) co-administration ameliorated Pb-adversely toxic alterations that impacted the cellular organization and activation of the apoptotic pathways in the testis (Al-Omair et al. 2017; Khodabandeh et al. 2021). Moreover, the oxidative injury was significantly recovered, and the antioxidant biomarkers such as GSH levels, SOD, and CAT activities were close to normal with co-administration of QCN, and the toxic symptoms following exposure to Al_2_O_3_NPs in mice were avoided (Shrivastava et al. 2014). Quercetin improved the oligospermia, male sex hormonal effects, and functional deficit induced by aluminum chloride and significantly reduced the degenerative testicular changes in Wistar rats (Olanrewaju et al. 2021). Thus, the current study demonstrates the hazardous effects of Al_2_O_3_NPs + Pb co-exposure along with their independent exposure and the role of QCN against that co-exposure in male rats.

## Materials and methods

### Chemicals and reagents

Gamma aluminum oxide nanoparticles (Al_2_O_3_NPs, M.W. = 101.96, less than 30-nm particle size) and QCN (C_15_H_10_O_7_, 2H_2_O, M.W. = 338.27) were obtained from Alpha Chemica (Mumbai, India). Lead acetate trihydrate (Pb CH_3_CO_2_)·2H_2_O, M.W. = 379.33, 99% purity) was obtained from Biochem Chemopharma (Cosne-Cours-Sur-Loire, France). All additional reagents/chemicals were obtained from Sigma Company and were of analytical class (St. Louis, MO).

### Animals and experimental design

Adult male Sprague–Dawley rats (*n* = 70) were bought from the National Research Center’s breeding section (Giza, Egypt). All rats were kept in well-ventilated, clean steel mesh cages with a 12-h light–dark cycle at 21–24 °C and 50–60% relative humidity. To keep the cages dry, wood-shaving bedding was used. Rats had unlimited access to tap water and regular rodent food throughout the experiment. Before testing, rats were given a 2-week acclimatization period to the experimental circumstances. The experimental protocol was authorized by Cairo University’s research committee on the ethics of animal use, with the reference number VETCU20092022460 which followed the general criteria of the National Institutes of Health Guide for the Care and Use of Laboratory Animals in Scientific Investigations. Every effort was made to treat the animals with compassion and to resolve ethical issues. Rats were weighed and assigned to seven groups at random (*n* = 10 each): G1: control group: received no treatment during the study time; G2: vehicle control group (corn oil): orally administered 2 ml/kg BW/day corn oil (Güleş et al. 2019); G3: QCN-treated group: orally administered 20 mg/kg BW/day QCN dissolved in 2 ml/kg BW corn oil (Farombi et al. 2012); G4: Al_2_O_3_NPs-exposed group: orally administered 100 mg Al_2_O_3_NPs/kg BW (Shumakova et al. 2015); G5: lead acetate exposed group (Pb): orally administered 50 mg Pb acetate/kg BW dissolved in distilled water (Mailafiya et al. 2020); G6: Al_2_O_3_NPs + Pb co-exposed group: co-administered Al_2_O_3_NPs and Pb at the previous mentioned doses; and G7: Al_2_O_3_NPs + Pb + QCN: co-administered Al_2_O_3_NPs, Pb, and QCN at the same previous doses.

All treatments were given orally via orogastric gavage once daily between 8 and 10 a.m. using a feeding needle for 60 days (16 gauge). In the case of Al_2_O_3_NPs, a new and fresh suspension was used every day. Before administering to the animals, we gave the Al_2_O_3_NPs suspensions a 15-min sonication in a distilled water bath using an ultrasonic cleaner (FRQ-1010HT, Hangzhou, China) and a 5-min vortex mix to ensure homogeneity.

Every week, all treatments were re-adjusted depending on the rats’ body weight changes. Pain, discomfort, damage, abnormal behavior, distress, mucous membrane color, breathing patterns, morbidity, and mortality were all closely monitored during the trial. The consumed amount of food and the weight of the animals were measured weekly.

### Sampling

All rats in each group were fasted overnight after the last dose, then weighed, and euthanized by cervical dislocation. Jugular vein blood samples were collected into a plain tube, allowed to clot at room temperature for 20 min, centrifuged for 10 min at 3000 rpm, and the resulting serum was stored at − 20 °C for later biochemical analysis. Then, rats were euthanized by cervical dislocations; the livers were collected, washed with physiological saline, and weighed. The liver specimens were divided into three sets. The first set was fixed in a 10% buffered neutral formalin solution for histopathological and histochemical investigation. The second one was used to prepare tissue homogenates for the assays defined below. The last one was kept at 4 °C till analysis of metal residues.

### Estimation of hepatic enzymes and bilirubin levels

Commercial kits using biodiagnostic kits, diagnostic and research reagents, Egypt to estimate serum alkaline phosphatase (ALP), alanine aminotransferase (ALT), and aspartate aminotransferase (AST) activities following the protocols of Reitmann (1957) and Kind (1954), respectively. The total and direct serum bilirubin was assessed based on the procedures of Walter and Gerade (1970) method.

### Lipid profile assay

Serum total cholesterol (TC) was assessed based on Kendall (1952) protocol. The triglycerides (TG) were evaluated consistently with the method of Bucolo and David (1973).The serum high-density lipoprotein cholesterol (HDL-C) concentration was estimated according to Assmann et al. (1983). While the serum concentrations of very low-density lipoprotein cholesterol (VLDL-C) and low-density lipoprotein cholesterol (LDL-C) were mathematically calculated following the method described by Friedewald et al. (1972).

### Oxidative stress and lipid peroxidation indicators in liver homogenate

The hepatic tissue contents of SOD Durak (1993), malondialdehyde (MDA) (Ohkawa et al. 1979), and GPx (Paglia and Valentine 1967) were estimated in liver homogenate using the colorimetric method using biodiagnostic kits, diagnostic and research reagents, Egypt.

### Histopathological examination

Each animal’s liver specimens were dissected and preserved in a 10% formalin solution for fixation. Then, they were dehydrated with ascending alcohol concentrations, cleared in xylene, embedded, and blocked in paraffin. After, sections of 4-μm thickness were taken and stained with hematoxylin and eosin (H&E) (Bancroft and Layton 2013). Samples of all rats were examined randomly with a light microscope (Olympus, Tokyo, Japan) at different magnifications and analyzed to find histological alterations.

### Histochemical examination

Periodic Acid Schiff’s stain (PAS) was performed to detect the glycogen in the cytoplasm of the hepatocytes (Layton et al. 2019). Masson’s trichrome stain was applied on liver tissue sections to demonstrate the distribution and quantity of collagen fibers among different treated groups.

### Histomorphometric measurements

Five sections from each group were randomly selected. From each section, two visual fields were photographed at high power, and the pictures were analyzed using a computerized Microsoft system. The size of hepatocytes, the nuclear diameter of hepatocytes, the size of binucleated hepatocytes, and the nuclear diameter of binucleated hepatocytes were estimated (Fazelipour et al. 2008).

### Determination of Al and Pb hepatic contents

Each liver sample was microwave-digested with 8 mL nitric acid and 1 mL of 30% hydrogen peroxide. Then, the Al and Pb contents were determined by an inductively coupled plasma–optical emission spectrometer (ICP-OES, model 5100, Agilent, Santa Clara, CA) with synchronous vertical dual view (SVDV). The intensity calibration curve was developed for each set of measurements using a blank and three or more Merck Company standards (Germany). Reference standards from Merck were used to verify the accuracy and precision of the metal measurements. A quality control sample containing known concentrations of trace elements from the National Institute of Standards and Technology (NIST) was used to validate the instrument’s results.

#### Statistical analysis

The data were analyzed with SPSS version 14 and one-way analysis of variance (ANOVA) (SPSS, Chicago, IL, USA). To compare means, Tukey’s multiple range test was used. A significance level of *p*-value < 0.05 was determined. A Shapiro–Wilk W test was used to ensure that all data were normally distributed.

## Results

### Effects on body weight change

The exposure to Al_2_O_3_NPs and/or Pb, significantly (*P* < 0.05) lessened the final body weight or weight gain compared to the control group. However, QCN co-treatment induced a significant (*P*<0.05) increase in the the final body weight and body weight gain compared to Al_2_O_3_NPs+Pb-co-exposed groups. Pb alone or in co-exposure with Al_2_O_3_NPs had no significant effect on the hepatosomatic index. On the contrary, the exposure to Al_2_O_3_NPs + Pb + QCN significantly (*P* < 0.05) reduced the hepatosomatic index relative to the Al_2_O_3_NPs+Pb-co-exposed group (Table 1).

**Table 1:** Effect of quercetin (QCN) oral dosing on body and liver weight change and hepatosomatic index of Sprague–Dawley rats exposed to aluminum trioxide nanoparticles (Al_2_O_3_NPs) and/or lead (Pb) for 60 days

Estimated parameters	Experimental groups						
	Control	CO	QCN	Al_2_O_3_NPs	Pb	Al_2_O_3_NPs + Pb	Al_2_O_3_NPs+Pb+QCN
Initial body weight (g)	182.00 ± 0.71	181.33 ± 3.79	180.67 ± 1.65	181.67 ± 0.62	182.00 ± 0.82	182.67 ± 1.03	184.33 ± 0.24
Final body weight (g)	215.00^a^ ± 2.55	209.67^a^ ± 5.20	213.00^a^ ± 7.63	192.67^b^ ± 1.65	194.67^b^ ± 2.36	195.00^b^ ± 0.71	218.00 ^a^ ± 0.82
Body weight change (g)	33.00^a^ ± 3.08	28.33^a^ ± 7.36	32.33^a^ ± 8.57	11.00^b^ ± 1.08	12.67^b^ ± 3.09	12.33^b^ ± 1.18	33.67 ^a^ ± 0.85
Liver weight (g)	6.46^a^ ± 0.27	6.37^a^ ± 0.33	6.57^a^ ± 0.08	5.30^c^ ± 0.07	6.53^a^ ± 0.05	6.17^ab^ ± 0.24	5.53 ^bc^ ± 0.37
Hepatosomatic index (%)	3.00 ^bc^ ± 0.09	3.03^abc^ ± 0.11	3.10 ^ab^ ± 0.14	2.75^cd^ ± 0.02	3.36^a^ ± 0.06	3.16 ^ab^ ± 0.11	2.54 ^d^ ± 0.16

Means within the same row carrying different superscripts are significantly different at *P* < 0.05. The values shown are means ± *SE*. *n* = 10

### Effects on liver function indicators

The serum hepatic enzymes (ALP, ALT, and AST) and bilirubin (total and directed) levels were significantly (*P* < 0.05) raised, following the single or combined exposure to Al_2_O_3_NPs and Pb for 60 days, compared to the control group (Table 2). Of note, the Al_2_O_3_NPs-exposed groups, even with QCN showed significant increases in total and direct bilirubin, compared to all other groups, including Pb-exposed groups. In contrast, the ALP, ALT, AST, total bilirubin, and direct bilirubin serum levels were significantly (*P *< 0.05) lower in the Al_2_O_3_NPs+Pb+QCN co-treated group than the Al_2_O_3_NPs and Pb-co-exposed group.

**Table 2:** Effect of quercetin (QCN) on serum levels of biochemical parameters of rats exposed to aluminum oxide nanoparticles (Al_2_O_3_NPs) and/or lead (Pb) for 60 days

Estimated parameters	Experimental groups						
	Control	CO	QCN	Al_2_O_3_NPs	Pb	Al_2_O_3_NPs + Pb	Al_2_O_3_NPs + Pb + QCN
AST(U/dL)	19.00^de^ ± 1.63	17.00^e^ ± 0.82	15.33^e^ ± 1.93	38.67^c^ ± 0.62	64.33^b^ ± 2.32	76.00^a^ ± 2.45	23.67^d^ ± 2.05
ALT (U/m)	5.33^c^ ± 0.62	5.00^c^ ± 0.41	6.00^c^ ± 0.41	15.00^a^ ± 0.71	15.33^a^ ± 0.85	15.67^a^ ± 1.03	11.33^b^ ± 0.85
ALP (U/dL)	46.33^b^ ± 9.58	44.33^b^ ± 4.09	41.67^b^ ± 2.46	67.67^a^ ± 11.61	74.00^a^ ± 6.04	76.00^a^ ± 1.41	60.00^ab^ ± 3.08
Total bilirubin (mg/dL)	1.08^b^ ± 0.26	1.02^b^ ± 0.03	0.68^bc^ ± 0.01	1.99^a^ ± 0.19	0.57^c^ ± 0.01	0.72^bc^ ± 0.02	1.71^a^ ± 0.11
Direct bilirubin (mg/dL)	2.01^bc^ ± 0.30	1.85^ cd^ ± 0.24	1.70^ cd^ ± 0.10	3.26^a^ ± 0.26	1.28^d^ ± 0.07	1.70^ cd^ ± 0.21	2.59^b^ ± 0.21
Total cholesterol (mg/dL)	108.33^d^ ± 3.06	99.00^d^ ± 7.08	69.00^e^ ± 1.22	150.33^c^ ± 7.26	182.67^b^ ± 4.25	206.33^a^ ± 7.00	112.00^d^ ± 2.04
Triglyceride (mg/dL)	80.67^c^ ± 2.66	78.67^c^ ± 2.87	47.00^e^ ± 1.47	94.33^b^ ± 7.32	103.33^b^ ± 4.64	130.00^a^ ± 2.16	62.67^d^ ± 2.66
LDL (mg/dL)	19.33^c^ ± 2.09	17.00^c^ ± 0.82	10.67^d^ ± 0.85	34.33^ab^ ± 2.25	35.33^ab^ ± 2.49	36.67^a^ ± 1.84	30.33^b^ ± 0.47
VLDL (mg/dL)	12.80^de^ ± 0.57	12.53^de^ ± 0.53	9.40^e^ ± 0.65	18.87^bc^ ± 2.10	20.67^b^ ± 1.73	26.00^a^ ± 0.43	15.73^ cd^ ± 1.39
HDL (mg/dL)	17.86^bc^ ± 1.76	17.67^bc^ ± 1.42	42.08^a^ ± 1.43	15.20^ cd^ ± 0.61	13.47^ cd^ ± 0.72	11.40^e^ ± 1.82	20.80^b^ ± 2.01

Means within the same row carrying different superscripts are significantly different at *P* < 0.05. The values shown are means ± *SE*. *n* = 10 group

*AST* aspartate aminotransferase; *ALT* alanine aminotransferase; *ALP* alkaline phosphatase; *LDL* low-density lipoprotein; *VLDL* very low-density lipoprotein; *HD* High-density lipoprotein

### Effects on lipid profile

Variations in the serum lipid profile figure of rats orally exposed to Al_2_O_3_NPs/or Pb for 60 days and those orally treated with QCN are displayed in Table 2. Compared with the control group, the serum levels of TC, TG, LDL, and VLDL were significantly (*P* < 0.05) increased in Al_2_O_3_NPs and/or Pb-exposed groups. Conversely, a significant decline in the HDL level was evident in rats of the Al_2_O_3_NPs and Pb co-exposed group relative to the control rats. A co-exposure to Al_2_O_3_NPs and Pb induced significant alterations in those parameters in an additive mechanism compared with rats exposed to those agents in solitary. On the contrary, QCN significantly (*P*< 0.05) improved the lipid profiles in the co-exposure (Al_2_O_3_NPs+Pb) group, with a very close figure to the control non-treated rats (Table 2).

### Effects on hepatic oxidative stress and lipid peroxidation indicators

**Fig. 1:**
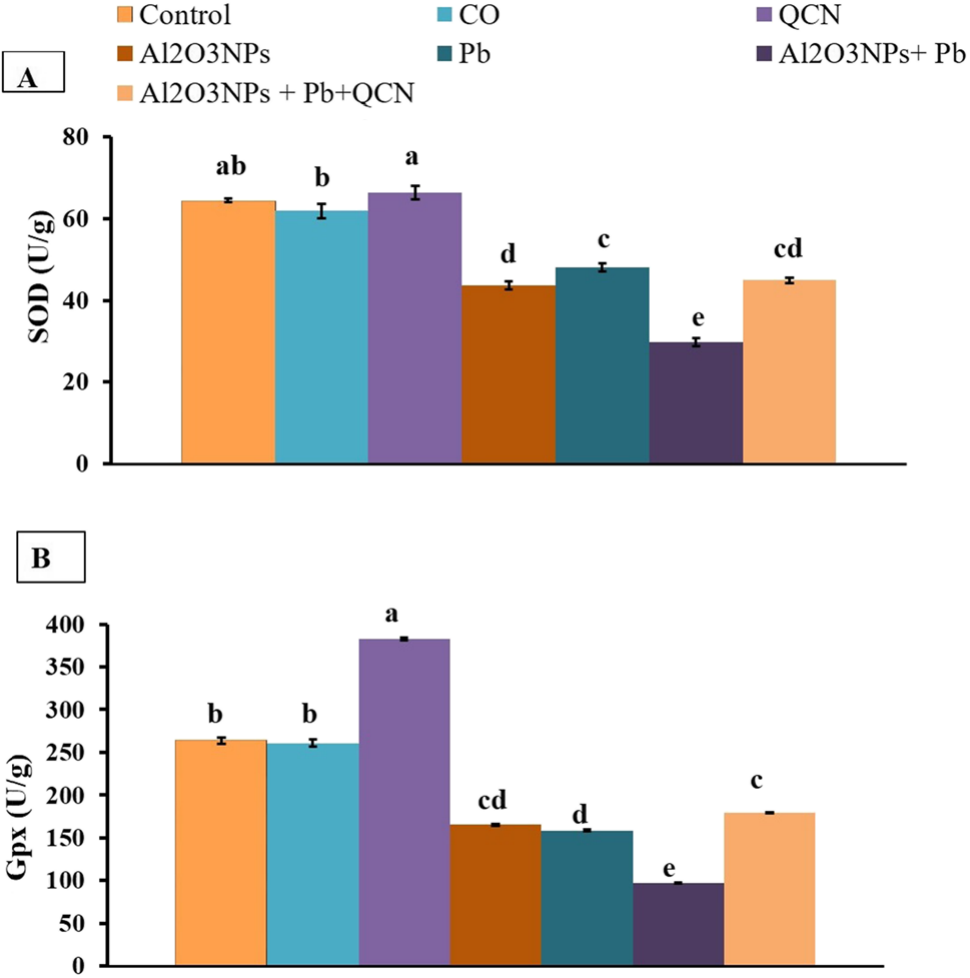
Effects of quercetin (QCN) on antioxidant enzymes (SOD (**A**) and GPx (**B**)) in liver homogenate of rats exposed to aluminum oxide nanoparticles (Al_2_O_3_NPs) and/or lead (Pb) for 60 days. Data are expressed as the mean ± SE (*n* = 10). Columns carrying different superscripts are significantly different (one-way ANOVA) (*P* < 0.05)

**Fig. 2:**
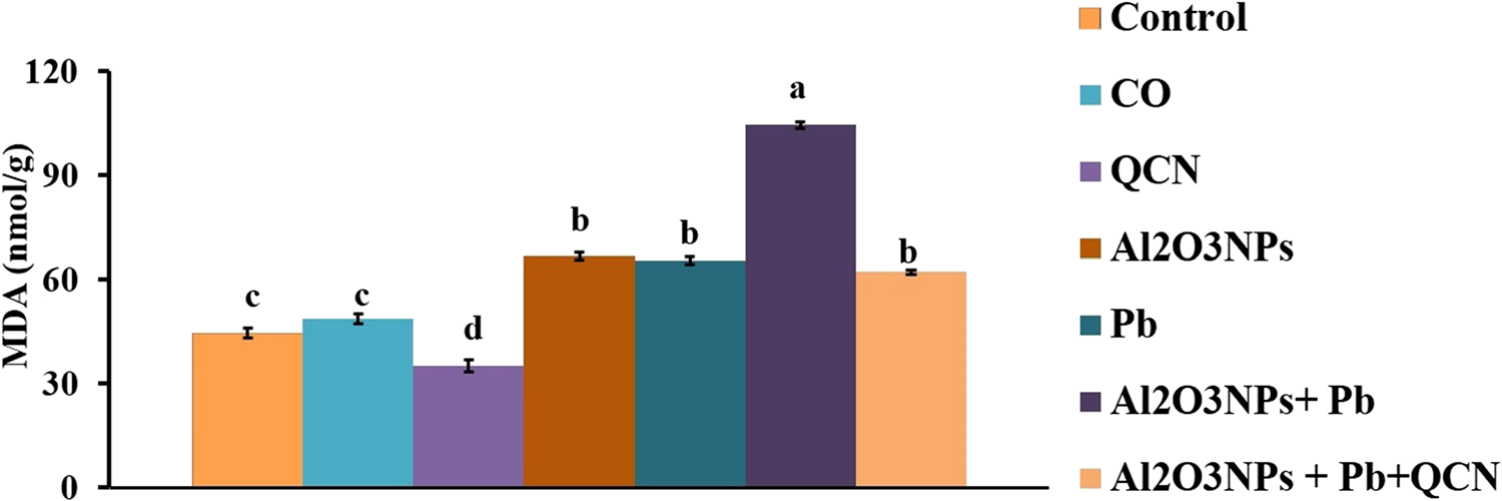
Effects of quercetin (QCN) on the lipid peroxidation indicator (MDA) in liver homogenate of rats exposed to aluminum oxide nanoparticles (Al_2_O_3_NPs) and/or lead (Pb) for 60 days. Data are expressed as the mean  ±  SE (*n* =  10). Columns carrying different superscripts are significantly different (one-way ANOVA) (*P*  < 0.05)

Regarding SOD, GPx, and MDA concentrations in liver homogenate, there were significant (*P* < 0.05) increase in GPx in the QCN group, while there was a significant (*P* < 0.05) decrease in hepatic MDA level relative to the control group (Figs. 1 and 2). In comparison with the control groups, Al_2_O_3_NPs, Pb, and Al_2_O_3_NPs + Pb groups showed a significant (*P* <0.05) reduction in SOD concentration (Fig. 1A). Hepatic GPx level decreased significantly (*P* < 0.05) in the Al_2_O_3_NPs, Pb, and Al_2_O_3_NPs + Pb groups, relative to the control group (Fig. 1B). Hepatic MDA increased significantly (*P* < 0.05) in the Al_2_O_3_NPs, Pb, and Al_2_O_3_NPs + Pb groups, relative to the control group (Fig. 2). Instead, the SOD and GPX levels increased significantly but MDA concentration decreased significantly (*P* < 0.05) in the Al_2_O_3_NPs+Pb+QCN group, relative to Al_2_O_3_NPs+Pb co-exposed group.

**Fig. 3:**
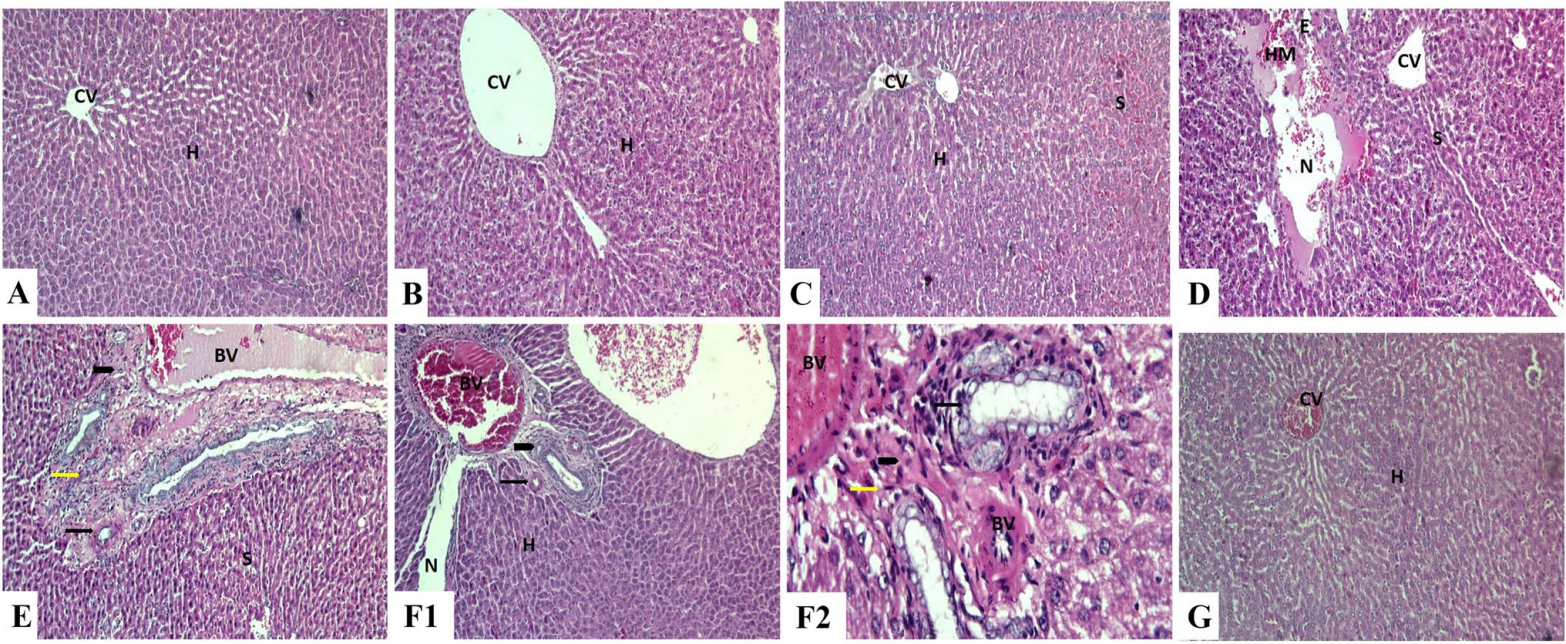
Liver sections of rat stained with H&E. Control (**A**), corn oil (**B**), and quercetin (**C**) groups showing a normal structure with normal hepatocytes in the hepatic cords and central vein (H&E,  × 100). **D**: Aluminum trioxide nanoparticle-treated group showing hepatocyte disorganization, congested central vein and blood sinusoids, necrotic area in hepatic parenchyma, hemorrhage, hemolysis, and edema (H&E, ×100). **E**: Lead acetate-treated group showing hepatocyte disorganization, congested blood vessels and sinusoids, vasculitis, and hypertrophy of blood vessel wall as well as newly formed bile ductules, fibrosis, and mononuclear leucocytic infiltrations in the portal area (H&E, ×100). **F1**: Co-exposure-treated group showing disorganization of hepatocytes, necrotic area along the hepatocytes, congested blood vessels, vasculitis, and hypertrophy of blood vessels wall associated with newly formed bile ductules and fibrosis in the portal area (H&E, ×100). **F2**: Co-exposure-treated group showing disorganized hepatocyte cords, hepatocyte vacuolations, necrosis with pyknotic nuclei, congested blood vessels and sinusoids, vasculitis, newly formed bile ductules, mononuclear leucocytic infiltrations (H&E, ×400). **G**: Co-exposure and quercetin-treated group showing normal hepatocytes in the hepatic cords and congested central vein (H&E, ×100). Abbreviations: H, hepatocytes; CV, central vein; S, sinusoid; N, necrotic area; HM, hemorrhage; E, edema; BV, blood vessel; arrow: bile ductules; arrowhead: portal area; yellow arrow: mononuclear leucocytic infiltrations

### Effects on Al and Pb hepatic residues

Relative to the control group, no detectable level of Pb was recorded in the hepatic tissues of all experimental groups except those exposed to Pb and those co-exposed to Al_2_O_3_NPs + Pb (Table 3). However, significantly (*P* < 0.05) lower hepatic Pb content was recorded in Al_2_O_3_NPs + Pb-co-exposed group relative to those singly Pb-exposed.

**Table 3:** Effect of quercetin (QCN) on liver content of lead (Pb) and aluminum (Al) of rats exposed to aluminum oxide nanoparticles (Al_2_O_3_NPs) and/or lead (Pb) for 60 days

	Experimental groups						
	Control	CO	QCN	Al_2_O_3_NPs	Pb	Al_2_O_3_NPs + Pb	Al_2_O_3_NPs + Pb + QCN
Al residues (ppm)	22.50^d^ ± 0.26	15.70^d^ ± 0.32	14.28^d^ ± 0.18	420.00^a^ ± 8.38	44.90^c^ ± 0.95	96.30^b^ ± 1.74	45.30^c^ ± 0.79
Pb residues (ppm)	ND	ND	ND	ND	0.63^a^ ± 0.04	0.12^b^ ± 0.01	ND

Means within the same row carrying different superscripts are significantly different at *P* < 0.05. The values shown are means ± *SE*. *n* = 10 group

*ND* not detected

Regarding Al accumulation in the hepatic tissue, the Al_2_O_3_NPs or Al_2_O_3_NPs + Pb-exposed groups had higher Al’s significant (*P* < 0.001) content, respectively, compared to the control group. Pb residue significantly decreased the Al content in the hepatic tissues, compared to the group independently administered Al. Moreover, the oral dosing of QCN significantly reduced the Al accumulation in hepatic tissues in the co-exposed rats.

### Histopathological and histomorphic findings

,The control, corn oil, and QCN groups showed a normal histological picture with normal hepatocytes and portal areas **(**Fig. 3A, B, C). On the contrary, Al_2_O_3_NPs and/or Pb, co-exposed groups revealed different pathological alterations. There was prominent vacuolar degeneration in the hepatocytes with focal coagulative necrotic changes surrounding the central vein. Binucleated hepatocytes were observed. The proliferation of Kupffer cells was also seen. Peri-central aggregations of mononuclear cellular inflammatory cells, particularly lymphocytes, were observed. There was congestion and dilatation of the central veins, portal veins, and blood sinusoids. Thickening of the wall of blood vessels with vasculitis and perivascular edema was noted. Focal hemorrhage with brown hemosiderin pigment deposition was observed among the hepatic parenchyma. Marked periportal fibrosis, hyperplastic bile duct epithelium, and newly formed bile ductules were seen in the portal area. The liver capsule showed thickening, subcapsular congestion, edema, and inflammatory cell infiltrations (Fig. 3D, E). The intensity of these alterations was more evident in co-exposure-treated group (Fig. 3F1, 2). Co-exposure with the QCN-treated group exhibited noticeable improvement in its histological picture, except for mild congestion in a few sections (Fig. 3G). The histomorphometric properties of hepatocytes and their nuclei between different treated groups compared with a normal untreated group are shown in Table 4.

**Table 4:** Effect of quercetin (QCN) on hepatic lesion score of rats exposed to aluminum trioxide nanoparticles (Al_2_O_3_NPs) and/or lead (Pb) for 60 days

Estimated parameters	Experimental groups						
	Control	CO	QCN	Al_2_O_3_NPs	Pb	Al_2_O_3_NPs + Pb	Al_2_O_3_NPs + Pb + QCN
Size of hepatocytes	12.33 ^b^ ± 0.85	12.67 ^b^ ± 1.18	12.67^b^ ± 0.62	11.00^bc^ ± 0.71	10.00^c^ ± 0.41	10.67^bc^ ± 0.47	16.33^a^ ± 0.24
Nuclear diameter of hepatocytes	6.67 ^a^ ± 0.24	6.33 ^a^ ± 0.24	6.67 ^a^ ± 0.24	5.33 ^b^ ± 0.24	5.00 ^b^ ± 0.00	5.00 ^b^ ± 0.00	6.33 ^a^ ± 0.47
Size of binucleated hepatocytes	13.33^c^ ± 0.24	13.33^c^ ± 0.24	13.33^c^ ± 0.24	15.33^bc^ ± 1.31	14.00^c^ ± 0.71	19.33 ^a^ ± 0.62	16.33 ^b^ ± 0.85
Nuclear diameter of binucleated hepatocyte	4.33 ^d^ ± 0.24	4.67^d^ ± 0.24	5.00^ cd^ ± 0.41	6.00^abc^ ± 0.41	6.33^b^ ± 0.62	7.00^a^ ± 0.41	5.33 ^bcd^ ± 0.47

Means within the same row carrying different superscripts are significantly different at *P* < 0.05. The values shown are means ± *SE*. *n* = 10 group

### Histochemical findings

**Fig. 4:**
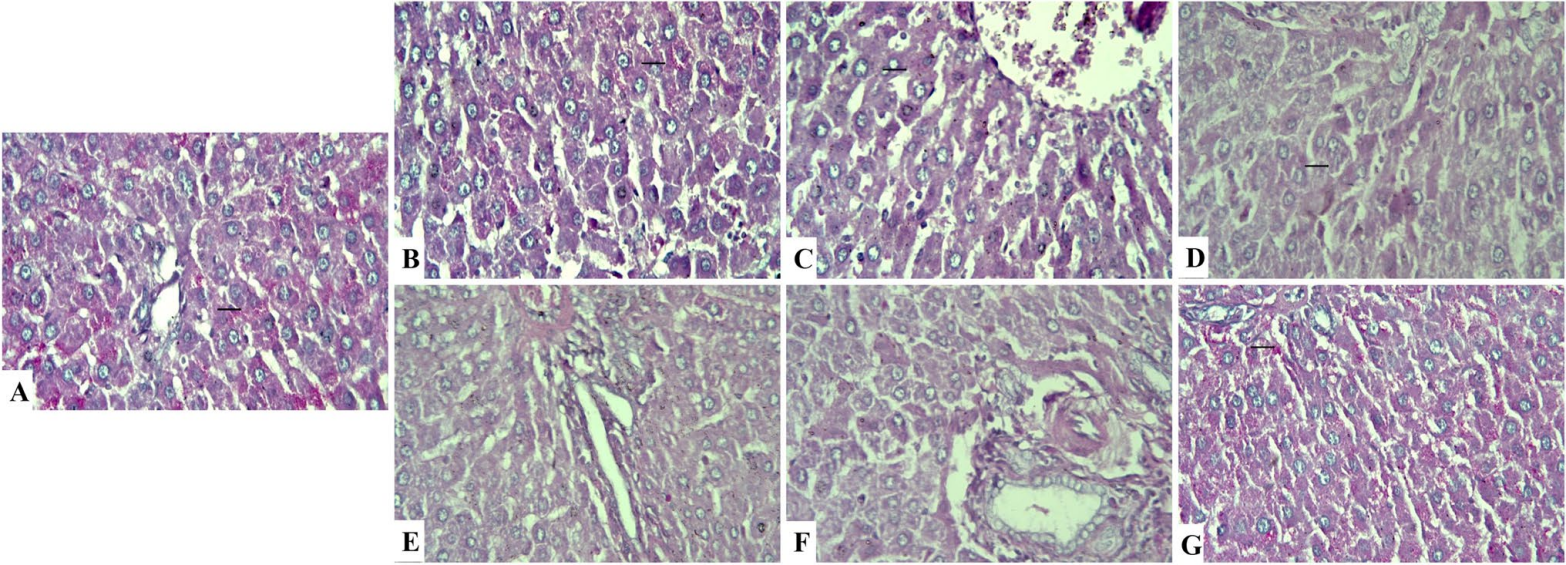
Liver sections stained with periodic acid Schiff’s stain that is identified by magenta color of glycogen granules in the hepatocyte cytoplasm (arrows). **A**: Control group showing strong PAS color of glycogen granules. **B**: Corn oil-treated group showing strong positive reaction. **C**: Quercetin-treated group showing strong PAS positive cells. **D**: Aluminum trioxide nanoparticle-treated group showing weak PAS results. **E**: Lead acetate-treated group showing decrease stain of cells. **F**: Co-exposure-treated group showing wide areas of negative reaction of stain. **G**: Co-exposure and quercetin-treated group showing increased glycogen content of cells (PAS, ×400)

**Fig. 5:**
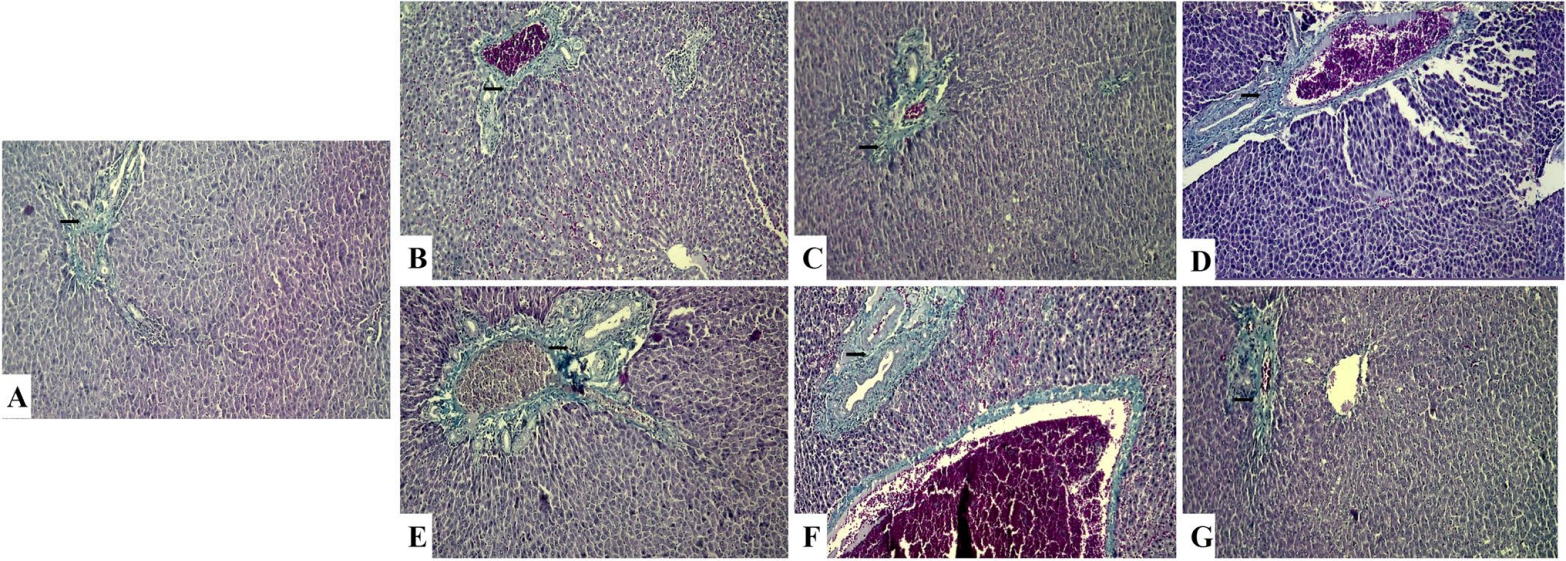
Liver sections stained with Masson trichrome that is identified by blue color of collagen fibers (arrows). **A**: Control group showing normal distribution of collagen fibers around central veins and in portal area. **B**: Corn oil-treated group showing normal reaction of stain. **C**: Quercetin-treated group showing normal stain of cells. **D**: Aluminum trioxide nanoparticle-treated group showing marked deposition of collagen fibers around central veins and in portal area. **E**: Lead acetate-treated group showing increase in collagen fibers. **F**: Co-exposure-treated group showing strong positive reaction. **G**: Co-exposure and quercetin-treated group showing normal distribution of collagen fibers (Masson trichrome, ×100)

Periodic Acid Schiff’s stain was applied on liver sections from all groups to demonstrate the glycogen content in the cytoplasm of the hepatocytes (Fig. 4). The magenta color of glycogen granules was more evident in control (Fig. 4A), corn oil (Fig. 4B), and QCN (Fig. 4C) as well as co-exposure with QCN-treated (Fig. 4G) groups. On the other side, Al_2_O_3_NPs (Fig. 4D), Pb (Fig. 4E), and co-exposure-treated (Fig. 4F) groups showed the weak color of glycogen granules. Masson trichrome staining for mature collagen fibers was also used, appearing as blue color (Fig. 5). The stain was absent in the liver of control (Fig. 5A), corn oil (Fig. 5B), and QCN (Fig. 5C)-treated groups, except around central veins and portal triads. Al_2_O_3_NPs (Fig. 5D), Pb (Fig. 5E), and co-exposure (Fig. 5F)-treated groups showed marked deposition of collagen fibers in pericentral and periportal areas. Treatment with Al_2_O_3_NPs and lead acetate with QCN (Fig. 5G) showed normal intensity and distribution of collagen fibers.

## Discussion

The co-exposure of Al_2_O_3_NPs with Pb has infrequently been investigated and is a chiefly indistinct issue, and the protective strategies also need more scientific clarification. Hence, the current research illustrated the hazardous effects of combined Al_2_O_3_NPs + Pb co-exposure along with their independent exposure and the role of QCN against that co-exposure in male rats. There are many factors affecting the Al_2_O_3_NPs toxicity as morphology, particle size, and dose. For the same metal NPs, smaller-sized NPs have existed more harmful effects than larger ones (Dong et al. 2019).

Our findings exhibited that Al_2_O_3_NPs and/or Pb exposure significantly decreased the final body weight or weight gain, compared to the control group. Similar effects were confirmed by Shumakova et al. (2015) as the co-administration of Al_2_O_3_NPs with Pb resulted in significant shifts in the relative body and organ weights. However, QCN induced a substantial increase in the body weight gain in Al_2_O_3_NPs and Pb-co-exposed group.

The significant single exposure to Al_2_O_3_NPs and mutual with Al_2_O_3_NPs + Pb + QCN (*P* < 0.05) reduced the hepatosomatic index relative to the control group. On the contrary, the hepatosomatic indices of fish treated with the three doses of aluminum sulfate were significantly higher (*P* < 0.05) higher than the control group with histopathological lesions in the hepatic tissues (Authman 2011).

Abdulkareem (2020) stated that QCN (20 mg/kg/day) had hepatoprotective efficacy and significantly (*P* < 0.05) decreased the hepatosomatic index, serum transaminases, and liver oxidative stress biomarker liver. The serum levels of hepatic enzymes (AST, ALT, and ALP) and bilirubin (total and directed) were significantly (*P* < 0.05) elevated following the single or combined exposure to Al_2_O_3_NPs and Pb for 60 days, compared to the control group. The transaminases, ALP, total bilirubin, and direct bilirubin levels were significantly improved in the Al_2_O_3_NPs + Pb + QCN-co-treated group to the Al_2_O_3_NPs and Pb-co-exposed group. Nanoparticles are likely to be accumulated in the liver and soft organs after their extensive distribution to the extravascular tissues (Sengul and Asmatulu 2020).

Hepatic necropsy showed cellular degeneration, necrosis, and congestion of sinusoidal blood vessels. Yousef et al. (2019) found that exposure to Al_2_O_3_NPs at an oral dose of 70 mg/kg for 75 days, significantly elevated the blood activities of ALP, AST, ALT, and bilirubin levels in rats. Moreover, the ingestion of Pb induced significant increases in ALT and AST activity although the total blood protein and albumin contents were significantly decreased (Ibrahim et al. 2012).

Our results showed that the serum levels of TC, TG, LDL, and VLDL were significantly increased in Al_2_O_3_NPs and/or Pb-exposed groups with a significant decline in the HDL level evident in rats of the two previous groups relative to the control rats. Similar findings were obtained by Abdou and Hassan (2014) who confirmed that Pb acetate exposure induced hyperlipidemia. Additionally, the authors stated that histopathological perturbations and DNA damage accompanied the biochemical disturbances resulting from Pb acetate. Moreover, El-Hussainy et al. (2016) recorded significant increases in serum levels of TG, total cholesterol, and LDL with a substantial decrease in serum HDL in the Al_2_O_3_NPs group. Lastly, Canli et al. (2017) documented that Al_2_O_3_NPs administrations for 2 weeks had significant alterations in the serum levels of cholesterol, glucose, bilirubin, triiodothyronine, triglyceride, estradiol, and immunoglobulin M in a dose-dependent mechanism in female rats.

Pretreatment with QCN normalized the TG, total cholesterol, and fractions and significantly increased HDL concentrations in hepato-intoxicated rats (Seiva et al. 2012). Our findings illustrated that the direct bilirubin (conjugated hyperbilirubinemia) level was significantly elevated in the Al_2_O_3_NPs group solitary. Evaluation of total bilirubin could denote hepatic or prehepatic jaundice; however, the rise in conjugated bilirubin proposes cholestasis or hepatocellular injury (Čvorović and Passamonti 2017).

Conjugated bilirubin is a biochemical indicator of hepatocellular dysfunction. While the catabolic product, bilirubin, is produced when the enzyme catalase and peroxidase break down the heme molecules in hemoglobin. Subsequently, in the liver, unconjugated bilirubin is converted to conjugated bilirubin by glucuronate (Harb and Thomas 2007). Our histopathological figures support the previous results as marked periportal fibrosis, hyperplastic bile duct epithelium, and newly formed bile ductules were seen in the portal area. The liver capsule showed thickening, subcapsular congestion, edema, and inflammatory cell infiltrations.

Cellular accumulation of Al_2_O_3_NPs resulted in interaction with cell components and binding with proteins and nucleic acids producing cellular oxidative damage. Also, cell death was caused by Al_2_O_3_NPs due to their ability to compromise mitochondrial membranes, deplete mitochondrial thiols, activate apoptotic caspases (9 and 3), and increase ROS production (Alshatwi et al. 2013; Elkhadrawy et al. 2021). Additionally, Liu et al. (2020) stated that Al_2_O_3_NPs toxicity induced apoptosis; subsequently, the expression of BCL-2, cyclin D1, Mdm2, and phospho-Rb was decreased and that of p53, Bax, p21, and Rb was increased.

In this respect, Morsy et al. (2016) and Li et al. (2020) found that the intraperitoneal injections of Al_2_O_3_NPs significantly decreased SOD and GPx levels, whereas the level of MDA was increased. While Canli and Canli (2020) stated that NPs reduced the hepatic SOD and CAT activities, but GPx activity showed no significant change in aquatics. This may be due to species variation in oxidative stress patterns.

Oxidative stress is a necessary consequence of Al-induced hepatic toxicity via nitric oxide formation, enhanced lipid peroxidation, and reduced cellular glutathione (Türkez et al. 2010). Zhang et al. (2021) recorded that Al_2_O_3_NPs induced hippocampal oxidative stress and inflammatory biomarkers such as IFN-γ, IL-1β, TNF-α, and IL-6 were significantly augmented in rats after a daily for 90 days. In this respect, exposure to metallic NPs has been reported to enhance ROS generation, oxidative stress, and subsequent damage to DNA (Sengul and Asmatulu 2020). Herein, QCN improved the hepatic antioxidant capacity and alleviated the hepatic tissue damage caused by Pb and Al_2_O_3_NPs. Similarly, QCN alleviated oxidative damage and apoptosis in Pb-poisoned chicken via the PI3K signaling pathway (Cai et al. 2021).

Various pathological alterations such as sinusoidal dilatation, lipid accumulation, congestion of the central vein, and lymphocyte infiltration were established in rat hepatic tissues after Al dosing for 1 month (Türkez et al. 2010). Previous histopathological examinations revealed dilatation of central veins, (Prabhakar et al. 2012), expansion of portal tracts, irregular disarray, necrosis of hepatocytes, and Kupffer cells, and congested blood sinusoids (Yousef et al. 2019). Moreover, degenerative changes, hepatic necrosis, congestion of sinusoidal blood vessel dilated central vein and expanded portal tract were observed (Morsy et al. 2016; Li et al. 2020). In addition, SA (2014) stated that lead acetate is a potent hepatotoxic pollutant, as it exists in an elevation in hepatic transaminase level, multiple histological changes in hepatocytes, liver parenchyma, and lymphocytic infiltration with significant DNA fragmentation in rats. Consequently, the histopathological alterations were exaggerated and more marked in the Al_2_O_3_NPs and Pb-co-exposed group. Al_2_O_3_NPs, Pb, and co-exposure-treated groups showed a few glycogen granules stained with Masson’s trichrome staining. Conversely, the liver of the QCN-treated group showed normal intensity and distribution of collagen fibers. The glycogen granule deposition in hepatocytes improved to be normal in the level of all QCN-treated groups relative to the control group, which was detected by PAS reaction.

Al_2_O_3_NPs and Pb-co-exposure resulted in some changes in liver tissue as hepatocyte necrosis and higher density of collagen fiber in the portal a, pericentral, and periportal areas. Collagenolytic activity may play an essential role in collagen buildup associated with hepatic fibrosis, and mammalian collagenase initiates collagen destruction throughout the fibrosis recovery phase (Roderfeld et al. 2007). Our results showed that QCN decreases glycogen accumulation within preneoplastic lesions and promotes its redistribution, a phenomenon also observed in models of hepatocellular carcinoma-induced liver fibrosis (Reyes-Avendaño et al. 2022). This evidence suggests that QCN reverses fibrogenesis induced by Al_2_O_3_NPs and Pb-co-exposure. It has been reported that the antifibrotic effect of QCN is mediated through the diminution of the myofibroblast population (Wu et al. 2017).

## Conclusion

Our results showed that QCN normalized serum lipid and oxidative stress profiles and minimized the Al_2_O_3_NPs and Pb co-toxic effects associated with its effective antioxidant properties. Moreover, it reduced the appearance of preneoplastic lesions and the accumulation of glycogen, and therefore fibrogenesis. Therefore, it is credible to recommend using QCN as an adjuvant remedy to NPs and potential HMs pollutants.

## Data Availability

All the data generated or analyzed during this study are included in this published article.

## References

[CR1] Abd-Elhakim YM, Hashem MM, Abo-EL-Sooud K, Hassan BA, Elbohi KM, Al-Sagheer AA (2021). Effects of co-exposure of nanoparticles and metals on different organisms: a review. Toxics.

[CR2] Abdou HM, Hassan MA (2014). Protective role of omega-3 polyunsaturated fatty acid against lead acetate-induced toxicity in liver and kidney of female rats. Biomed Res Int.

[CR3] Abdulkareem SMNN (2020). Ameliorating potential of quercetin on liver function, genotoxicity and oxidative damage induced by 2,3,7,8-tetrachlorodibenzo-p-dioxin in liver of male rats. Pak J Zool.

[CR4] Al-Omair MA, Sedky A, Ali A, Elsawy H (2017). Ameliorative potentials of quercetin against lead-induced hematological and testicular alterations in Albino rats. Chin J Physiol.

[CR5] Albishtue AA, Almhanna HK, Yimer N, Zakaria MZA, Haron AW, Almhanawi BH (2020) Effect of edible bird's bird’s nest supplement on hepato-renal histomorphology of rats exposed to lead acetate toxicity. Jordan J Biol Sci 13:213–218

[CR6] Alshatwi AA, Subbarayan PV, Ramesh E, Al-Hazzani AA, Alsaif MA, Alwarthan AA (2013). Aluminium oxide nanoparticles induce mitochondrial-mediated oxidative stress and alter the expression of antioxidant enzymes in human mesenchymal stem cells. Food Addit Contam Part A Chem Anal Control Expo Risk Assess.

[CR7] Assmann G, Schriewer H, Schmitz G, Hägele E (1983). Quantification of high-density-lipoprotein cholesterol by precipitation with phosphotungstic acid/MgCl2. Clin Chem.

[CR8] Authman MM (2011). Environmental and experimental studies of aluminium toxicity on the liver of Oreochromis niloticus (Linnaeus, 1758) fish. Life Science Journal.

[CR9] Bancroft J, Layton C (2013). The hematoxylins and eosin. Bancroft’s theory and practice of histological techniques. Elsevier.

[CR10] Bucolo G, David H (1973). Quantitative determination of serum triglycerides by the use of enzymes. Clin Chem.

[CR11] Cai P, Zhu Q, Cao Q, Bai Y, Zou H, Gu J, Yuan Y, Liu X, Liu Z, Bian J (2021). Quercetin and allicin can alleviate the hepatotoxicity of lead (Pb) through the PI3K signaling pathway. J Agric Food Chem.

[CR12] Canli E, Canli M (2020). Effects of aluminum, copper and titanium nanoparticles on the liver antioxidant enzymes of the Nile fish (Oreochromis niloticus). Energy Rep.

[CR13] Canli EG, Atli G, Canli M (2017). Response of the antioxidant enzymes of the erythrocyte and alterations in the serum biomarkers in rats following oral administration of nanoparticles. Environ Toxicol Pharmacol.

[CR14] Čvorović J, Passamonti S (2017). Membrane transporters for bilirubin and its conjugates: a systematic review. Front Pharmacol.

[CR15] Dong L, Tang S, Deng F, Gong Y, Zhao K, Zhou J, Liang D, Fang J, Hecker M, Giesy JP, Bai X, Zhang H (2019). Shape-dependent toxicity of alumina nanoparticles in rat astrocytes. Sci Total Environ.

[CR16] Durak I (1993). A methodological approach to superoxide dismutase (SOD) activity assay based on inhibition of nitroblue tetrazolium (NBT) reduction. Clin Chim Acta.

[CR17] el El-Hussainy HM, Hussein AM, Abdel-Aziz A, El-Mehasseb I (2016). Effects of aluminum oxide (Al2O3) nanoparticles on ECG, myocardial inflammatory cytokines, redox state, and connexin 43 and lipid profile in rats: possible cardioprotective effect of gallic acid. Can J Physiol Pharmacol.

[CR18] Elkhadrawy B, Abou-Zeid S, El-Borai N, El-Sabbagh H, El-Bialy BE-S (2021). Potential toxic effects of aluminum nanoparticles: an overview. Journal of Current Veterinary Research.

[CR19] Farombi E, Adedara I, Akinrinde S, Ojo O, Eboh A (2012). Protective effects of kolaviron and quercetin on cadmium-induced testicular damage and endocrine pathology in rats. Andrologia.

[CR20] Fazelipour S, Kiaei S, Tootian Z, Dashtnavard H (2008). Histomorphometric study of hepatocytes of mice after using heroin. Int J Pharmacol.

[CR21] Friedewald W, Levy RI, Fredrickson DS (1972). Estimation of the concentration of low-density lipoprotein cholesterol in plasma, without use of the preparative ultracentrifuge. Clin Chem.

[CR22] Güleş Ö, Kum Ş, Yıldız M, Boyacıoğlu M, Ahmad E, Naseer Z, Eren Ü (2019). Protective effect of coenzyme Q10 against bisphenol-A-induced toxicity in the rat testes. Toxicol Ind Health.

[CR23] Hamza RZ, Al-Juaid NS, Althubaiti EH (2018). Antioxidant effect of carnosine on aluminum oxide nanoparticles (Al2O3-NPs)-induced hepatotoxicity and testicular structure alterations in male rats. Int J Pharmacol.

[CR24] Harb R, Thomas DW (2007). Conjugated hyperbilirubinemia: screening and treatment in older infants and children. Pediatr Rev.

[CR25] Ibrahim NM, Eweis EA, El-Beltagi HS, Abdel-Mobdy YE (2012). Effect of lead acetate toxicity on experimental male albino rat. Asian Pac J Trop Biomed.

[CR26] Kendall FE (1952). A simplified method for the estimation of total cholesterol in serum and demonstration of its specificity. J Biol Chem.

[CR27] Khodabandeh Z, Dolati P, Zamiri MJ, Mehrabani D, Bordbar H, Alaee S, Jamhiri I, Azarpira N (2021). Protective effect of quercetin on testis structure and apoptosis against lead acetate toxicity: an stereological study. Biol Trace Elem Res.

[CR28] Kind J (1954). Determination of the activity of alkaline phosphatase. J Clin Path.

[CR29] Layton C, Bancroft JD, Suvarna SK (2019) 4—Fixation of tissues. In: Suvarna SK, Layton C, Bancroft JD (eds) Bancroft’s theory and practice of histological techniques, 8th edn. Elsevier, pp 40–63

[CR30] Li H, Huang T, Wang Y, Pan B, Zhang L, Zhang Q, Niu Q (2020). Toxicity of alumina nanoparticles in the immune system of mice. Nanomedicine (lond).

[CR31] Liu H, Zhang W, Fang Y, Yang H, Tian L, Li K, Lai W, Bian L, Lin B, Liu X, Xi Z (2020). Neurotoxicity of aluminum oxide nanoparticles and their mechanistic role in dopaminergic neuron injury involving p53-related pathways. J Hazard Mater.

[CR32] Mailafiya MM, Abubakar K, Chiroma SM, Danmaigoro A, Rahim EBA, Moklas MAM, Zakaria ZAB (2020). Curcumin-loaded cockle shell-derived calcium carbonate nanoparticles: a novel strategy for the treatment of lead-induced hepato-renal toxicity in rats. Saudi Journal of Biological Sciences.

[CR33] Morsy GM, El-Ala KSA, Ali AA (2016). Studies on fate and toxicity of nanoalumina in male albino rats:lethality, bioaccumulation and genotoxicity. Toxicol Ind Health.

[CR34] Offor SJ, Mbagwu HO, Orisakwe OE (2017). Lead induced hepato-renal damage in male albino rats and effects of activated charcoal. Front Pharmacol.

[CR35] Ohkawa H, Ohishi W, Yagi K (1979). Colorimetric method for determination of MDA activity. Biochemistry.

[CR36] Olanrewaju JA, Akinpade TG, Olatunji SY, Owolabi JO, Enya JI, Adelodun ST, Fabiyi SO, Desalu AB (2021). Observable protective activities of quercetin on aluminum chloride-induced testicular toxicity in adult male Wistar rat. Journal of Human Reproductive Sciences.

[CR37] Paglia DE, Valentine WN (1967). Studies on the quantitative and qualitative characterization of erythrocyte glutathione peroxidase. J Lab Clin Med.

[CR38] Prabhakar PV, Reddy UA, Singh SP, Balasubramanyam A, Rahman MF, Indu Kumari S, Agawane SB, Murty US, Grover P, Mahboob M (2012). Oxidative stress induced by aluminum oxide nanomaterials after acute oral treatment in Wistar rats. Journal of Applied Toxicology : JAT.

[CR39] Reitmann S (1957). Colorimetric method for the determination of serum glutamic pyruvate and glutamic oxaaloacetate transaminase. Amer J Clin Path.

[CR40] Reyes-Avendaño I, Reyes-Jiménez E, González-García K, Pérez-Figueroa DC, Baltiérrez-Hoyos R, Tapia-Pastrana G, Sánchez-Chino XM, Villa-Treviño S, Arellanes-Robledo J, Vásquez-Garzón VR (2022) Quercetin regulates key components of the cellular microenvironment during early hepatocarcinogenesis. Antioxidants (Basel, Switzerland) 11(2):358 10.3390/antiox11020358 PMC8868318 35204240

[CR41] Roderfeld M, Hemmann S, Roeb E (2007). Mechanisms of fibrinolysis in chronic liver injury (with special emphasis on MMPs and TIMPs). Z Gastroenterol.

[CR42] Zi SA (2014). Hepatotoxic effects of lead acetate in rats: histopathological and cytotoxic studies. J Cytol Histol.

[CR43] Seiva FR, Chuffa LG, Braga CP, Amorim JP, Fernandes AA (2012). Quercetin ameliorates glucose and lipid metabolism and improves antioxidant status in postnatally monosodium glutamate-induced metabolic alterations. Food and Chemical Toxicology : an International Journal Published for the British Industrial Biological Research Association.

[CR44] Sengul AB, Asmatulu E (2020). Toxicity of metal and metal oxide nanoparticles: a review. Environ Chem Lett.

[CR45] Shrivastava R, Bhargava R, Flora S (2014). Antioxidant activity and free radical scavenging potential of alpha lipoic acid and quercetin against Al2O3 nanoparticle-induced toxicity in mice. Free Radicals and Antioxidants.

[CR46] Shumakova A, Trushina E, Mustafina O, SKh S, Gmoshinsky I, Khotimchenko S,  (2015). Lead toxicity in its joint administration with the aluminium oxide nanoparticles to rats. Vopr Pitan.

[CR47] Türkez H, Yousef MI, Geyikoglu F (2010). Propolis prevents aluminium-induced genetic and hepatic damages in rat liver. Food and Chemical Toxicology : an International Journal Published for the British Industrial Biological Research Association.

[CR48] Walter M, Gerade R (1970). Bilirubin direct/total. Microchem J.

[CR49] Wu L, Zhang Q, Mo W, Feng J, Li S, Li J, Liu T, Xu S, Wang W, Lu X, Yu Q, Chen K, Xia Y, Lu J, Xu L, Zhou Y, Fan X, Guo C (2017). Quercetin prevents hepatic fibrosis by inhibiting hepatic stellate cell activation and reducing autophagy via the TGF-β1/Smads and PI3K/Akt pathways. Sci Rep.

[CR50] Yousef MI, Mutar TF, Kamel MAE-N (2019). Hepato-renal toxicity of oral sub-chronic exposure to aluminum oxide and/or zinc oxide nanoparticles in rats. Toxicol Rep.

[CR51] Zhang H, Jiao W, Cui H, Sun Q, Fan H (2021). Combined exposure of alumina nanoparticles and chronic stress exacerbates hippocampal neuronal ferroptosis via activating IFN-γ/ASK1/JNK signaling pathway in rats. J Hazard Mater.

