# Correction to: Ameliorative effects of quercetin against hepatic toxicity of oral sub‑chronic co‑exposure to aluminum oxide nanoparticles and lead‑acetate in male rats

**DOI:** 10.1007/s00210-022-02369-2

**Published:** 2022-12-17

**Authors:** Khaled Abo-EL-Sooud, Yasmina M. Abd-Elhakim, Mohamed M. M. Hashem, Abeer E. El-Metwally, Bayan A. Hassan, Hayat H. M. El-Nour

**Affiliations:** 1 Department of Pharmacology, Faculty of Veterinary Medicine, Cairo University, Giza, Egypt; 2 Department of Forensic Medicine and Toxicology, Faculty of Veterinary Medicine, Zagazig University, Zagazig, Egypt; 3 Pathology Department, Animal Reproduction Research Institute, Giza, Egypt; 4 Pharmacology Department, Faculty of Pharmacy, Future University, Cairo, Egypt; 5 Biology of Reproduction Department, Animal Reproduction Research Institute, Giza, Egypt

**Correction to: Naunyn-Schmiedeberg’s Archives of Pharmacology**

10.1007/s00210-022-02351-y

In the published version, the name of the last author contained an error.

The correct name of the last author should be **Hayat H.M. El-Nour**.

The original article has been corrected.

